# Fertility Knowledge Among Women Struggling to Conceive Without Medical Intervention: A Brief Report

**DOI:** 10.3389/fgwh.2022.828052

**Published:** 2022-02-11

**Authors:** Maria Halleran, Andie Chernoff, Jennifer L. Gordon

**Affiliations:** Department of Psychology, University of Regina, Regina, SK, Canada

**Keywords:** fertility awareness, fertility knowledge, education, fertile window, infertility

## Abstract

**Introduction:**

Approximately 1 in 6 women experience infertility. Though medical treatments for infertility exist, they are very costly and highly burdensome for women. It is therefore desirable to optimize women's chances of conception without medical intervention by ensuring that they have adequate knowledge of the female menstrual cycle and the timing of the fertile window. The current study therefore aimed to assess the degree to which women struggling to conceive without medical intervention are knowledgeable about these topics.

**Methods:**

One hundred and two women of reproductive age (18–45 years old) in Canada and the United States who had been struggling to conceive without medical intervention for ≥12 months completed an online survey including a questionnaire assessing knowledge related to reproduction and fertility.

**Results:**

Mean accuracy score on the Fertility Knowledge Questionnaire was 67%. Seventy-two women were not aware that the week before ovulation was associated with the highest chances of conception. Women using cervical mucus tracking to increase chances of conception were more knowledgeable (*p* = 0.02), as were women with more formal education (*p* = 0.01). Conversely, women who had been attempting to conceive for longer had lower fertility knowledge (*p* = 0.03). Age, number of children, and family income were unrelated to fertility knowledge (*p* > 0.05).

**Discussion:**

Our findings suggest that women who are struggling to conceive would benefit from education related to the timing and identification of the fertile window. Reproductive and primary healthcare providers can play an important role in assessing fertility knowledge and addressing knowledge gaps to improve chances of successful conception.

## Introduction

One in 6 reproductive-aged couples experiences infertility (1), defined as being unable to achieve pregnancy despite ≥12 months of focused attempts to conceive. While most research on infertility focuses on women or couples undergoing assisted reproductive technologies (ART), such as *in vitro* fertilization or intrauterine insemination, less than half of all women with infertility ever pursue ART (2, 3), either because of financial constraints or personal preference. Among women who do pursue ART, stress is the most commonly-cited reason for premature discontinuation (4). Remarkably, this is even true in countries where fertility treatments are government-funded: one study in Sweden found that of 450 couples offered three government-funded IVF cycles, 54% did not complete all three *in vitro* fertilization cycles despite not achieving a pregnancy, with “psychological burden” being the most-commonly cited reason for prematurely discontinuing treatment (5).

Given the lack of access as well as the high financial and emotional burden of ART, it is important that women attempting to conceive be armed with the necessary knowledge to maximize their chances of conceiving without the need for medical intervention. One important component of this knowledge is an understanding of a woman's fertile window in relation to other events of the menstrual cycle. Research has identified the 5 days prior to ovulation, in addition to the day of ovulation itself, as days during which conception could occur if unprotected intercourse were to occur (6, 7). In contrast, no case of conception has ever been documented more than 24 h *after* ovulation (8). Importantly, research furthermore identifies the 2 days before ovulation as the days of highest fertility, rather than the day of ovulation itself (6, 7, 9). In fact, the most recent study suggests that the day of ovulation is associated with a considerable drop in fertility relative to the days leading up to it (9). However, the extent to which this is known among women with infertility who are struggling to conceive without medical intervention remains unknown. The current study therefore aimed to assess fertility knowledge, including accurate knowledge of the fertile window, in this population.

## Methods

### Participants

Reproductive aged women (18–45 years) from across Canada and the United States were recruited to participate in an online study examining the relationship between psychological coping strategies and infertility-related mood (10), advertised *via* social media. To qualify, women had to report having difficulty achieving pregnancy over the past 12 or more months, despite active attempts to conceive. They were also required to be planning to actively attempt to conceive during their next menstrual cycle. The only exclusion criterion was current use of ART, including *in vitro* fertilization, intrauterine insemination, and use of ovulation-enhancing medications. Individuals were compensated a $15.00 Amazon e-gift card for their participation in the baseline survey. All participants provided informed consent by reading an online consent form and clicking “I agree” prior to completing the survey. This study was reviewed and approved by the University of Regina Research Ethics Board (#2018-032).

### Procedure

While the entire study in which participants enrolled lasted one full menstrual cycle, the current study was a cross-sectional examination of self-reported fertility knowledge assessed at baseline. Prospective participants were instructed to make initial contact with researchers *via* Facebook messenger to express their interest in the study. The researcher then emailed a link to an online survey assessing study eligibility. Women deemed eligible to participate were invited to provide their contact information. The researcher then contacted these women, provided an overview of the study design, and obtained written consent to participate. Consenting women were sent a link to an online survey.

The survey included a section about demographic and reproductive health history. Participants were asked to report their age, relationship status, ethnicity and were asked to complete questions about reproductive health history, including previous pregnancies, miscarriages, abortions, fertility treatments, infertility-related diagnoses, and the fertility confirming methods they used to increase chances of conceiving. Additionally, twelve items assessing knowledge about basic human fertility were administered, including items pertaining to the fertile window. These questions were inspired by a “Fertility Knowledge Quiz” included on a governmental patient education website but is no longer publicly available. Specific items and response options are listed in Table 2 and Supplementary Table 1.

### Statistical Analyses and Power Calculations

Descriptive statistics were used to examine participant characteristics and responses on the Fertility Knowledge Questionnaire. Pearson correlations were also used to examine the relationship between various baseline characteristics, such as education and reproductive history, and percentage of accurate responses on the Fertility Knowledge Questionnaire. Chi square analyses were also used to examine the use of various fertility tracking methods as predictors of responses to particular items on the Fertility Knowledge Questionnaire.

As this was a secondary analysis of data collected for another purpose, power calculations were conducted as sensitivity analyses. Using G^*^Power, it was determined that a sample of 102 allowed for the detection of a small correlation of *r* = 0.27 with 80% power, setting alpha at 0.05. For Chi-square analyses, the largest critical z score was calculated to be 1.96, setting power at 80% and alpha at 0.05. This value was lower for fertility tracking methods that were more moderately endorsed, however.

## Results

### Participant Characteristics

While 1,006 women completed the eligibility survey, only 559 were deemed eligible to participate. Of those, 105 consented to participate and completed the baseline questionnaire. Data from 3 participants were excluded due to suspicious answer patterns, and data from the remaining 102 participants were analyzed. The demographic and reproductive characteristics of these women are reported in Table 1. Consistent with our exclusion of women pursuing (and who can afford) ART, our sample reported mean individual and household incomes well below the national averages for either Canada or the United States. The large majority had at least a high school diploma, though only 19% had a university degree. Participants reported that they had been actively trying to conceive between 12 and 156 months. More than half of the women had no live children and rates of prior miscarriage were high. When asked to report the strategies used to improve chances of conceiving, tracking one's menstrual cycle and timing intercourse during their fertile window were the most endorsed strategies, followed by using ovulation predictor tests, and measuring basal body temperature to confirm ovulation (Table 1).

**Table 1:** Participant characteristics.

	**Mean (SD) or %**
**Demographic characteristics**
Mean age (*SD*)	28.2 (6.2)
Race/ethnicity	
White/Caucasian	69%
Black/African-American	16%
Hispanic/Latina	11%
Other	4%
Education level	–
Some high school education	12%
High school diploma	35%
Some college education	34%
Bachelor's degree	13%
Master's degree	6%
Mean individual annual income	$20,000–$34,999
Mean annual household income	$35,000–$49,999
**Reproductive health characteristics**
Months trying to conceive (SD)	35.3 (34.0)
Number of current children	–
None	62%
1	19%
2+	19%
Previous pregnancy	64%
Previous miscarriage	58%
Prior fertility treatment	13%
Diagnosis of endometriosis	9%
Diagnosis of polycystic ovarian syndrome (PCOS)	25%
Irregular menstruation or acne without PCOS diagnosis	23%
Strategies for trying to conceive	
Cycle tracking	82%
Timed intercourse during “fertile window”	67%
Ovulation predictor tests	47%
Monitoring cervical mucus	40%
Basal body temperature measurement	20%
Nothing	7%
Mean number of conception strategies used	2.7 (1.5)

### Fertility Knowledge

Responses to the Fertility Knowledge Questionnaire are reported in Table 2. Mean percentage accuracy score was *M* (*SD*) = 67 (18). Although most women knew that the day of ovulation is not the only day during which conception could occur, it appeared that the majority of women (72%) were not aware that the week *before* ovulation was associated with the highest chances of conception. Conversely, only 22% of respondents were correct in identifying the week *after* ovulation as being associated with the lowest chances of conceiving. In examining conception method endorsement as a predictor of responses to the question about the week following ovulation, endorsement of cervical mucus tracking was significantly associated with a greater likelihood of correct responses (χ^2^ = 5.2, *p* = 0.02) such that 38% of women endorsing mucus tracking responded correctly to this answer, vs. 17% of women who did not. Pearson correlations revealed that respondent years of education was positively correlated with overall fertility knowledge quiz scores (*r* = 0.25, *p* = 0.01). A greater number of months spent trying to conceive was also correlated with lower fertility knowledge (*r* = −0.22, *p* = 0.03). Age, number of children, and family income were unrelated to fertility knowledge (*p* > 0.05).

**Table 2:** Responses to fertility awareness quiz.

	**True**			**False**	
Pregnancy can happen the very first time a person has sex.	95%			5%	
A woman will get pregnant only if she has sex on the same day she ovulates.	12%			88%	
At age 17, men become fertile, which means they can get a woman pregnant.	30%			70%	
Cervical secretions are one sign a woman is fertile.	80%			20%	
The size of a man's penis affects his ability to get a woman pregnant.	0%			100%	
It is normal for women to have menstrual cycles that are shorter or longer than 28 days.	90%			10%	
After having a baby, a woman can only get pregnant again when her periods return.	9%			91%	
When a woman gets pregnant, what determines whether she will have a boy or a girl?	**1**	**2**		**3**	
1—The father's sperm 2—The mother's egg 3—Neither, it is random	69%	1%		30%	
Which of the following is not a sign of ovulation?	**1**	**2**		**3**	**4**
1– Increase in body temperature 2– Monthly period 3– Slight ache or pain in abdomen (near ovary) 4– Cervical secretions	11%	69%		20%	1%
Rank each response on the scale below from *least likely (1)* to *most likely (5)* to lead to pregnancy:	**1**	**2**	**3**	**4**	**5**
Having unprotected sex the week after ovulation	23%	26%	20%	11%	21%
Having unprotected sex the day of ovulation	2%	4%	11%	18%	66%
Having unprotected sex the week before ovulation	5%	10%	28%	26%	30%

*Gray cells indicate answers considered correct*.

## Discussion

Basic fertility knowledge appeared to be relatively high in our sample of women struggling to conceive. For example, the majority of women were aware that intercourse did not have to take place on the day of ovulation for conception to occur and were able to recognize that a woman could have regular menstrual cycles in the absence of ovulation. However, there appeared to be confusion regarding the timing of a woman's fertile window in relation to ovulation: specifically, only 1 in 4 women recognized the week before ovulation as being associated with peak fertility. Women endorsing use of cervical mucus monitoring were more likely to have accurate knowledge related to the timing of a woman's fertile window and women with more education were more accurate in their fertility test scores overall.

This misunderstanding regarding the timing of the fertile window may have important consequences for women trying to conceive without medical intervention, especially women who are not using cervical mucus monitoring. In the current sample, nearly half of women endorsed the use of ovulation predictor tests, which identify the surge in luteinizing hormone (LH) that precedes ovulation by ~12–36 h. Importantly, however, a positive urine test is often only obtained 12 h after the true LH surge (11), leaving little to no time to time intercourse before ovulation takes place. Among women relying on this method, the belief that fertility increases *after* ovulation could result in women missing the fertile window altogether, thereby increasing the time to successful conception. The observation that women who had been trying to conceive for a longer time were less knowledgeable about women's fertility may suggest that a lack of knowledge contributes to decreased chances of conception with each cycle, perhaps due to a failure to appropriately time intercourse during the fertile window. Future research prospectively recording acts of intercourse and the timing of ovulation will be well suited to confirming this proposed mechanism of action. These findings suggest that education about the fertile window by a healthcare provider would be of value for women expressing a desire to conceive.

To our knowledge, three prior studies have assessed fertility knowledge among North American women pursuing ART. In one survey, 37% of women were deemed to have a moderate to high degree of fertility awareness based on their knowledge of the signs of ovulation, such as cervical mucus and basal body temperature increases (12). A second study of ART patients estimated that 26% of women had what was deemed to be adequate knowledge of the fertile window (13). These study findings are relatively consistent with our finding that 30% of women in the current study correctly identified the week leading up to ovulation as the time of highest fertility. In contrast, though, a Canadian study found that 76% of women pursuing ART had adequate knowledge of the fertile window. Like our study, though, they observed a significant negative correlation between years of formal education and fertility knowledge test scores (14), which would be consistent with the lower knowledge level in the current study given the lower socioeconomic status of our sample. Thus, our findings extend these earlier studies by focusing on a population that is largely understudied in the area of infertility: women with infertility but who are not intending to pursue ART, a sample that is likely to be socioeconomically disadvantaged compared to women with the financial means of pursuing fertility treatments. Despite this socioeconomic disadvantage, our sample was similarly knowledgeable about the fertile window compared to previously studied patient samples, perhaps because a large proportion of women in our study were actively monitoring their cervical mucus to track their fertility.

These findings should be interpreted in light of a few limitations. First, more details might have been asked regarding the sources of women's knowledge about human fertility. Second, while the current study examined the cross-sectional relationship between fertility knowledge and length of time spent trying to conceive, a longitudinal study of fertility knowledge, fertility-related behaviors, and time to conception, would better clarify the extent to which accurate fertility knowledge influences conception success. Third, there was a fairly small number of women (102) who consented to participate, despite 1,006 women completing the eligibility survey. Finally, we were unable to examine potential differences in knowledge between Canadians and Americans as participants were not asked to specify which of the two countries they lived in.

## Conclusion

While women struggling to conceive without medical intervention appear to have generally adequate fertility knowledge, there appears to be some misinformation regarding the exact timing of the fertile window, which may impede women's efforts at conception. Healthcare providers can play an important role in providing education to women attempting to conceive regarding the importance of timing intercourse in the week leading up to suspected ovulation to optimize chances of conceiving. Women using ovulation predictor tests, especially, would benefit from the knowledge that the day of ovulation is associated with a significant decline in fertility and that intercourse should therefore precede a positive test to optimize conception success. At a public health level, primary and reproductive healthcare providers can play an important role in assessing fertility knowledge and educating women about how to optimize chances of conception without medical intervention.

## Data Availability Statement

The raw data supporting the conclusions of this article will be made available by the authors, without undue reservation.

## Ethics Statement

The studies involving human participants were reviewed and approved by University of Regina Research Ethics Board. The patients/participants provided their written informed consent to participate in this study.

## Author Contributions

MH wrote the first draft of the manuscript. AC conceptualized the idea, collected the data, and edited the final draft. JG conceptualized the idea, supervised data collection, conducted data analysis, provided funding for the research, and provided critical edits to the final manuscript. All authors contributed to the article and approved the submitted version.

## Funding

This research was supported by the Saskatchewan Health Research Foundation Grant 3791. JG was supported by a Tier II Canadian Institutes of Health (CIHR) Canada Research Chair. MH was also supported by a Canada Graduate Scholarship from the Social Sciences and Humanities Research Council (SSHRC).

## Conflict of Interest

The authors declare that the research was conducted in the absence of any commercial or financial relationships that could be construed as a potential conflict of interest.

## Publisher's Note

All claims expressed in this article are solely those of the authors and do not necessarily represent those of their affiliated organizations, or those of the publisher, the editors and the reviewers. Any product that may be evaluated in this article, or claim that may be made by its manufacturer, is not guaranteed or endorsed by the publisher.
