# Supplementary material for: Fertility Knowledge Among Women Struggling to Conceive Without Medical Intervention: A Brief Report
Source: Front Glob Womens Health. 2022 Feb 11;3:828052. doi: 10.3389/fgwh.2022.828052 (PMC8873180; doi:10.3389/fgwh.2022.828052)
Supplement: Supplementary file 1 [file Table_1.DOCX]

|  | **True** | | | **False** | |
| --- | --- | --- | --- | --- | --- |
| Pregnancy can happen the very first time a person has sex. |  | | |  | |
| A woman will get pregnant only if she has sex on the same day she ovulates. |  | | |  | |
| At age 17, men become fertile, which means they can get a woman pregnant. |  | | |  | |
| Cervical secretions are one sign a woman is fertile. |  | | |  | |
| The size of a man’s penis affects his ability to get a woman pregnant. |  | | |  | |
| It is normal for women to have menstrual cycles that are shorter or longer than 28 days. |  | | |  | |
| After having a baby, a woman can only get pregnant again when her periods return. |  | | |  | |
| When a woman gets pregnant, what determines whether she will have a boy or a girl?  1 – The father’s sperm  2 – The mother’s egg  3 – Neither, it is random | **1** | **2** | | **3** | |
|  |  |  | |  | |
| Which of the following is not a sign of ovulation?   1. Increase in body temperature 2. Monthly period 3. Slight ache or pain in abdomen (near ovary) 4. Cervical secretions | **1** | **2** | | **3** | **4** |
|  |  |  | |  |  |
| Rank each response on the scale below from *least likely (1)* to *most likely (5)* to lead to pregnancy: | **1** | **2** | **3** | **4** | **5** |
| Having unprotected sex the week after ovulation |  |  |  |  |  |
| Having unprotected sex the day of ovulation |  |  |  |  |  |
| Having unprotected sex the week before ovulation |  |  |  |  |  |

**Supplementary Table 1**. Fertility Knowledge Quiz
